# The role of adverse childhood experiences in predicting child abuse perpetration among married mothers in Alexandria, Egypt: a cross-sectional study

**DOI:** 10.1186/s12905-024-02903-9

**Published:** 2024-01-23

**Authors:** Yasmine Yousry Mohammed, Mervat Wagdy Abu-Nazel, Reham Said Ibrahim Aly, Zeinab Nazeeh Shata

**Affiliations:** 1 Family Health Department, High Institute of Public Health, Alexandria University, 165 El Horreya Avenue – El Hadara, Alexandria, Egypt; 2 Ministry of Health and Population, Alexandria, Egypt

**Keywords:** Adverse childhood experiences (ACEs), Child abuse, Child maltreatment, Domestic violence, Spousal violence, Married mothers, Parenting practices, Egypt

## Abstract

**Background:**

Adverse childhood experiences (ACEs) are receiving increased amounts of attention as a critical public health issue. ACEs have a massive impact on future violence victimization and perpetration. They are also associated with lifelong mental and physical health consequences as well as premature mortality. The present study aimed to investigate the role of different ACEs among married mothers in predicting the risk of child abuse perpetration in offspring.

**Methods:**

A cross-sectional study was conducted on 350 mothers of children aged 2–12 years attending family health centres in Alexandria. The mothers completed a predesigned interview questionnaire on sociodemographic data and data related to ACEs, spousal violence, and child abuse perpetration. Suitable bivariate and multivariate statistical tests were used to analyse the collected data using version 20.0 of the Statistical Package for Social Sciences (SPSS).

**Results:**

Two-thirds of mothers (66.3%) reported ever having been exposed to any ACE, and 18.6% of them had experienced 3 or more ACEs. Psychological abuse (46%) and witnessing domestic violence (17%) were the most common. Psychological aggression (95.4%), minor physical assault (79%), and neglect (52%) were the most common forms of child abuse perpetrated by the mothers. The number of ACEs experienced by mothers showed a moderate positive significant correlation with the 5 forms of child abuse examined. Different ACEs, mother’s age, socioeconomic status, and current exposure to spousal violence were found to be independent predictors of different forms of child abuse (psychological aggression, neglect, minor physical assault, and severe physical assault).

**Conclusion:**

Different practices of family violence are strongly connected throughout different stages of an individual’s life and across generations. Further understanding of the interconnections among forms of violence and addressing them should be prioritized. Additionally, concerted national strategies across all levels and sectors are needed to address this complex problem.

**Supplementary Information:**

The online version contains supplementary material available at 10.1186/s12905-024-02903-9.

## Background

Childhood experiences impact people’s health throughout their life course. During the past decade, adverse childhood experiences (ACEs) have been increasingly regarded as an international public health concern [1]. ACEs are potentially traumatic events that occur in childhood (0–17 years) and include experiencing violence, abuse, or neglect; witnessing violence in the home; and having a family member attempt or die by suicide [2]. 

In 2015, a Public Health Wales ACE survey conducted on 2,000 adult males and females aged 18–69 years revealed that 46.5% of participants had experienced at least one ACE before the age of 18 years, while 13.6% of them had experienced four or more ACEs. The rates of ACEs ranged from 4.6% for living with a drug-using household member to 22.8% for experiencing more than one episode of verbal abuse [1]. 

Research evidence has indicated that ACEs are associated with negative long-term health and behavioural outcomes [1, 3–6]. Exposure to childhood adversity has an impact on adult physical and mental health, increasing the risk of chronic diseases such as heart disease and cancer, depression, suicide, and posttraumatic stress disorder [3–5]. Higher rates of ACEs are associated with high-risk behaviours, including smoking, heavy drinking, substance abuse, poor diet, and early unintended pregnancy [1, 3, 4]. 

In addition, ACEs increase the risk of violence victimization, perpetration and intergenerational transmission of violence [1, 6]. Bellis et al. suggested that people who experience ACEs as children often end up raising their own children in households where ACEs are more common [1]. Such a cycle of childhood adversity can lock successive generations of families into poor health and antisocial behaviour for generations [7]. A 2014 UNICEF study reported that approximately three-quarters of children (76.3%) were corporally punished, which was significantly predicted by their mothers’ childhood history of negative rearing experience and poor interparental relationships [8]. 

In Egypt, child abuse is evidently an escalating multifaceted problem. In 2018, severe physical disciplinary practices reached 45% [9], whereas earlier estimates reached 43% in 2014 [10]. During the past few decades, stressful living conditions such as low income status, overcrowding and cultural and religious misconceptions that normalize violent disciplinary practices have contributed to the intergenerational transmission of violence against children [10–12]. Moreover, Egyptian mothers are more forced than fathers to use these violent disciplinary practices because they believe that people will think they are unable to control their children’s misbehaviours if they do not [10, 12]. Evidence from the 2014 Egyptian Demographic and Health Survey (EDHS) revealed that mothers who were exposed to ACE of witnessing spousal violence were 2–3 times more likely to slap, strike and shout at their children compared to mothers who were not exposed to such ACE [7]. 

While most related research on the impact of ACEs on women’s health and well-being has focused on adults’ physical and mental health consequences, few studies have investigated parenting practices, the transgenerational continuity of violence and the relationship between maternal ACEs and child behaviour [6, 13–17]. Additionally, in 2016, the World began to implement the “2030 Agenda for Sustainable Development”, where ending all forms of violence against women and children are regarded as core targets for achieving sustainable development goals [18]. 

The present study hypothesizes that a maternal childhood history of adversity can predict child abuse perpetration in offspring. There is a paucity of studies investigating this issue in general and in Arab countries in particular. The current study aimed to investigate the role of different ACEs among married mothers in predicting the risk of child abuse perpetration in offspring in Alexandria, Egypt.

## Methods

### Study design and setting

This was a facility-based cross-sectional study that was conducted between 2017 and 2018. The study was carried out in 3 family health centres (FHCs) out of 11 centres affiliated to the Ministry of Health in Alexandria. The chosen centres, Elsyoof, Sanstefano and El-Amrawy, represented those with the highest attendance rates.

### Study population

The study involved currently married mothers of children aged 2–12 years attending family medicine clinics affiliated to the abovementioned FHCs. Mothers who were not living with their children, were divorced/separated, or were widowed were excluded.

### Study sample

Based on a previously published report by the World Health Organization [19], the rate of violence perpetration among those who experienced abuse during their childhood ranged from 8 to 40%. Using StatsDirect software [20], and 24% as an average rate of violence perpetration with a power of 80%, a 95% confidence interval and an error of 0.05, the minimum estimated sample size was 281 mothers.

The final sample size of mothers who agreed to participate in the study was 350 mothers, who were equally allocated among the three selected FHCs. Each FHC was visited 4 days a week to randomly recruit women who fulfilled the study eligibility criteria. Each visited centre had a daily attendance list for clients. Every other woman in the list was approached to give her consent to join the study after explanations of the study objectives and discussion of the consent form. This process was repeated until the allocated sample size was completed.

### Data collection tools

The data were collected through face-to-face interviews using the following tools:

1. A pre-coded structured interview questionnaire was used to collect personal and sociodemographic data, including age, age at marriage, duration of marriage, number of children, residence, and mothers’ and husbands’ education and occupation. The socioeconomic level was categorized according to an Egyptian tool designed by Fahmy and El-Sherbini to assess the socioeconomic level within Egyptian culture [21]. The cut-off points for the socioeconomic score were as follows: very low (< 16), low (16-<21), moderate (21-<26), and high [26–31].
2. A predesigned questionnaire that included 12 different childhood adversities based on a review of previous ACE studies was used to assess mothers’ exposure to ACEs (Appendix I) [1, 22]. The survey included five items that received four Likert scale responses (never, seldom, sometimes and frequently) and assessed ACEs related to physical and emotional abuse by a family member, witnessing domestic violence, sexual abuse by older person (5 or more years older), and gender discrimination by a family member. In addition, seven items were given dichotomous responses (yes/no) and included history of parental separation/divorce and death; household substance abuse; and a family member with a history of mental illness, chronic disease, suicide attempt, or imprisonment. For dichotomous calculations of exposure to physical abuse, emotional abuse, and domestic violence, never/seldom responses (not exposed) = 0, while sometimes/always (exposed) = 1. However, regarding exposure to sexual violence, never (not exposed) = 0, while seldom/sometimes/frequently (exposed) = 1.
3. The Arabic version of the Conflict Tactics Scales Parent‒Child (CTSPC) [23] was used to assess ***child maltreatment/ child abuse.*** It comprises 3 scales, namely, non– violent discipline (4 items); psychological aggression (5 items), and physical assault (5 items for corporal punishment, 4 items for severe physical assault, and 4 items for very severe physical assault). In addition, two supplemental scales were used to assess neglect (5 items) and sexual abuse (2 items). In the present study, the sexual abuse scale was excluded because previous research in the Egyptian community revealed that mothers were not sexual abuse perpetrators for their children [24, 25]. The proposed responses for the abovementioned items were “never”, ” yes, but not during the last month”, “yes, once or twice during the last month” and “yes, more than twice during the last month”. The Arabic version of the CTS-PC was scored as follows: “0” for the “never” response, “1” for “yes, but not during the last month”, “2” for “yes, once or twice during the last month”, and “3” for “yes, more than twice during the last month”. A total subscale was calculated for each of the child abuse forms (psychological aggression, neglect, minor physical assault, severe physical assault, and very severe physical assault). For dichotomous calculations of each type of child abuse, mothers who scored > 0 on the relevant total subscale were considered positive for the type.
  The total child abuse score was calculated by adding total scores of the following subscales: psychological aggression, minor physical assault, severe physical assault, very severe physical assault, and neglect. Internal consistency testing was performed for the different subscales, and the Cronbach’s alpha values were as follows: minor physical assault = 0.63, severe physical assault = 0.85, psychological aggression = 0.67, neglect = 0.49 and very severe physical assault = 0.35.
4. The Arabic version of the HITS (Hurt, Insult, Threaten, Scream) tool [26] was used to assess mothers’ current experience of spousal violence. It consists of 4 questions, each scored from 1 to 5, yielding a total score of 20. A cut-off score > 10 was used to identify women who were victimized by their husbands. Mothers who scored > 10 on the HITS were labelled as currently exposed to spousal violence.

### Statistical analysis

The data were collected by a member of the research team. The collected data were revised for completeness and accuracy and then coded, computed, and cleaned. Statistical analysis was performed using the Statistical Package for Social Sciences (SPSS version 20.0). In the bivariate analysis, a *p*-value of less than 0.05 was considered to indicate statistical significance, and significant variables were retained in the multivariate analysis. Multivariate analysis was conducted through 5 logistic regression models to detect predictors of the types of child abuse perpetrated by mothers, where the outcomes of the five regression models were psychological aggression, neglect, minor physical assault, severe physical assault, and very severe physical assault. Accordingly, adjusted odds ratios with 95% confidence intervals (CIs) were calculated.

### Ethical consideration

This study was approved by the Ethics Committee of the High Institute of Public Health, Alexandria University. Eligible women who gave their verbal consent to participate were included in the study after being clearly informed about the study objective and possible harms and benefits of participation. Additionally, confidentiality and privacy were clearly assured.

## Results

The sociodemographic characteristics of the 350 mothers who provided consent to participate in the present study are illustrated in Table 1. The mean age of the mothers was 30.4 ± 5.6 years, while the number of children ranged from 1 to 6, with a mean of 2.3 ± 1.4 children. More than one-quarter of them (95; 27.1%) were university graduates, and three-quarters (263; 75.2%) were housewives.

**Table 1:** Respondents’ sociodemographic characteristics

Characteristic (*N* = 350)	Level	n (%)
Age (in years)	20–29	160 (45.7)
	30–39	162 (46.3)
	40-	28 (8.0)
Mean (SD) 30.4 ± 5.6		
Age of marriage (in years)	Less than 25	246 (70.3)
	25–29	82 (23.4)
	30+	22 (6.3)
Mean (SD) 22.6 ± 3.6		
Duration of marriage (in years)	Less than 5	145 (41.4)
	5–10	122 (34.9)
	More than 10	83 (23.7)
Mean (SD) 7.8 ± 4.5		
Number of children Mean (SD) 2.3 ± 1.4	1–2 children	220 (62.9)
	3–6 children	130 (37.1)
Residence	Urban	349 (99.7)
	Rural	1 (0.3)
Education of the mother	Illiterate or can read and write	18 (5.2)
	Compulsory education	61 (17.4)
	Secondary education	176 (50.3)
	University and above	95 (27.1)
Education of the husband	Illiterate or can read and write	6 (1.6)
	Compulsory education	26 (7.5)
	Secondary education	157 (44.9)
	University and above	161 (46.0)
Occupation of the mother	Housewife	263 (75.2)
	Clerk	42 (12.0)
	Professional	33 (9.4)
	Craftswomen	12 (3.4)
Occupation of the husband	Doesn’t work/unemployed	23 (6.6)
	Clerk	121 (34.6)
	Professional	80 (22.9)
	Craftsman	63 (18.0)
	Trader	38 (10.8)
	Farmer	25 (7.1)
Socioeconomic level	Very low	26 (7.4)
	Low	98 (28.0)
	Moderate	159 (45.5)
	High	67 (19.1)

As shown in Table 2, approximately two-thirds of the sampled mothers (232; 66.3%) reported ever having been exposed to any ACE. Emotional abuse, witnessing domestic violence and physical abuse were the most commonly reported forms of ACEs experienced by mothers (160; 45.7%, 61; 17.4% and 46; 13.1%, respectively), with a mean of 1.37 ± 1.43 ACE*s.* The mothers commonly perpetrated different forms of child abuse, ranging from 76 (21.4%) for very severe physical assault to 334 (95.4%) for psychological aggression. Concerning mothers’ current exposure to spousal violence, the sampled mothers had a mean HITS score of 9.56 ± 2.70, with approximately one-third of them (102; 29.1%) having HITS scores > 10 denoting current exposure to spousal violence.

**Table 2:** Adverse childhood experiences (ACEs), child abuse perpetration, and current spousal violence victimization among the sampled mothers

Characteristic (*N* = 350)	Level	*n* (%)
Number of ACEs	No ACE	118(33.7)
	One ACE	105 (30.0)
	Two ACEs	62 (17.7)
	Three ACEs or more	65 (18.6)
Types of ACEs experienced by the mothers in their childhood	Emotional abuse	160 (45.7)
	Witnessing domestic violence	61 (17.4)
	Physical abuse	46 (13.1)
	Sexual abuse by an older person	42 (12)
	Household substance abuse	41 (11.7)
	Family member with chronic disease	32 (9.1)
	Gender discrimination	30 (8.6)
	Divorced parents	30 (8.6)
	Dead parent	24 (6.9)
	Family member with mental health problem	19 (5.4)
	Family member attempt suicide	11 (3.1)
	Family member imprisoned	9 (2.6)
Types of child abuse perpetrated by the mothers	Psychological aggression	334 (95.4)
	Minor physical assault	276 (79.0)
	Neglect	182 (52.0)
	Severe physical assault	110 (32.0)
	Very severe physical assault	76 (21.4)
Current exposure to spousal violence based on the Hurt, Insult, Threaten, and Scream (HITS) score	Negative (≤ 10)	248 (70.9)
	Positive (> 10)	102 (29.1)

**Fig. 1:**
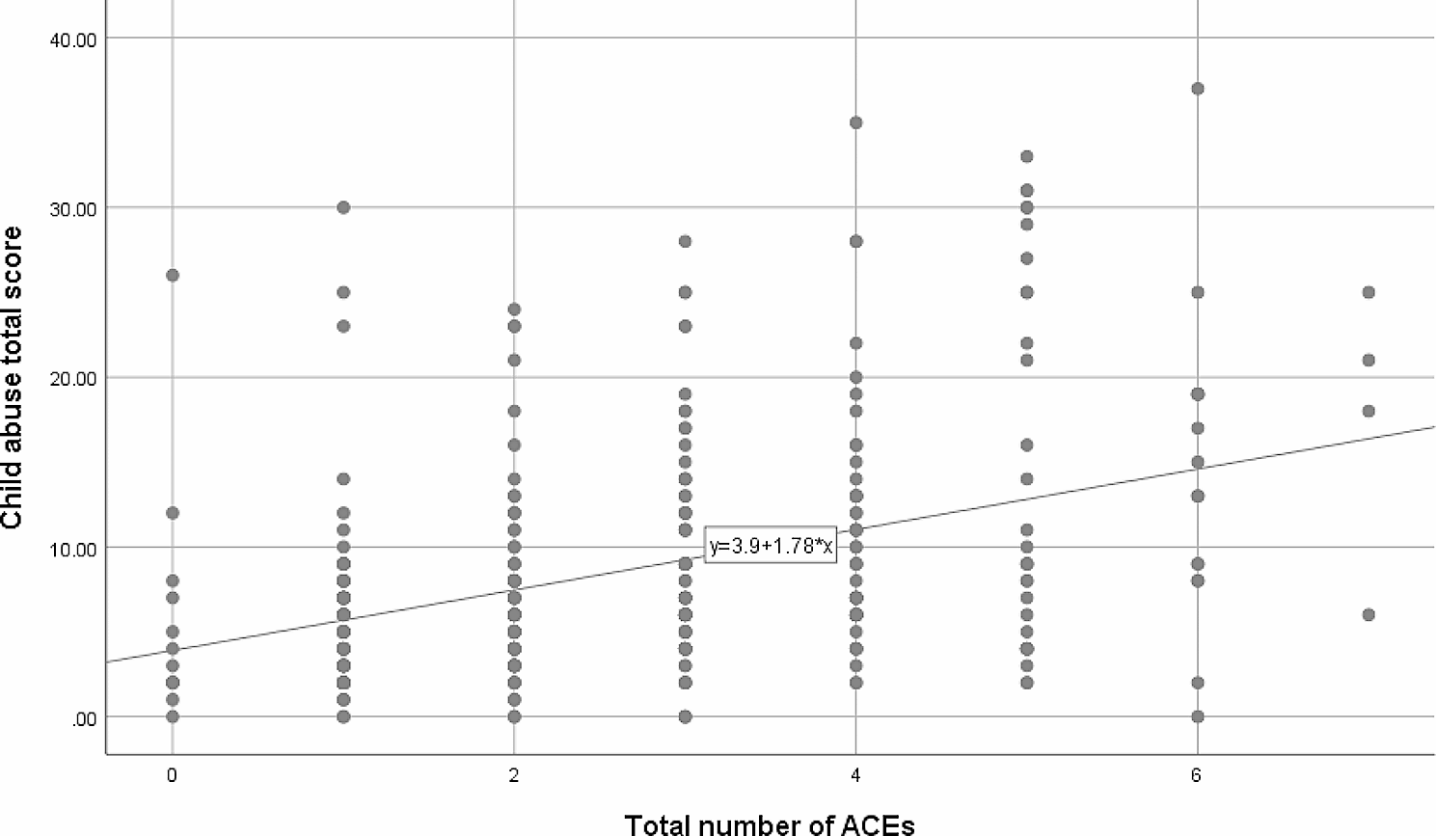
Correlation between the number of ACEs mothers experienced and their total child abuse scores Pearson correlation coefficient (r): 0.394, *p* < 0.001

The correlations between the number of ACEs experienced by the studied mothers and their total and subscale scores on the CTS-PC for child abuse are illustrated in Table 3; Fig. 1. A moderately positive significant correlation was revealed between the number of ACEs experienced by the studied mothers and their reported child abuse scores on psychological aggression (*r* = 0.339, *p* < 0.001), minor physical assault (*r* = 0.333, *p* < 0.001), severe physical assault (*r* = 0.302, *p* < 0.001) and very severe physical assault (*r* = 0.339, *p* < 0.001). Moreover, a weak significant positive relationship was detected for neglect (*r* = 0.225, *p* < 0.001). The dose‒response relationship is further illustrated in Fig. 1, where an increase in the number of ACEs reported by mothers was associated with an increase in their total child abuse scores (*r* = 0.394, *p* < 0.001).

**Table 3:** Correlations between the total number of ACEs mothers were exposed to and their scores on the different child maltreatment subscales

	Child maltreatment Total	Psychological aggression	Neglect	Minor physical assault	Severe physical assault	Very severe physical assault
Total number of ACEs (r) *	0.394	0.339	0.225	0.333	0.302	0.339
*p*-value	< 0.001	< 0.001	< 0.001	< 0.001	< 0.001	< 0.001

*: Pearson correlation coefficient

Five regression models were used to predict different types of child abuse perpetrated by mothers (Table 4). According to our bivariate analysis, 7 variables were significantly associated with mothers’ practices of ***psychological aggression***. However, only mothers’ ACE related to emotional abuse and mother’s older age were found to be independent predictors of mothers’ perpetration of psychological aggression toward children [OR = 5.8; 95% CI (1.4–24.6) and OR = 1.1; 95% CI (1.0-1.3), respectively].

**Table 4:** Predictors of different forms of child abuse perpetrated by mothers towards their children (multivariate analysis results):

Variables	Adjusted OR^a^	95% CI^b^	*p*-value
**Psychological aggression**			
ACE of physical abuse	1.8	0.5–7.2	0.413
ACE of emotional abuse	5.8	1.4–24.6	0.018*
ACE of witnessing domestic violence	0.6	0.2–2.6	0.537
ACE of household substance abuse	1.6	0.2–13.4	0.687
Current exposure to spousal violence	2.1	0.6–8.3	0.276
Older age of the mother	1.1	1.0-1.3	0.026*
Low socioeconomic status	0.6	0.4–7.8	0.682
Very low socioeconomic status	0.3	0.1-2.0	0.229
High socioeconomic status	1.1	0.1–2.8	0.423
**Neglect**			
ACE of witnessing domestic violence	1.1	0.7–1.7	0.846
ACE of family member with mental problem	3.4	1.1–10.7	0.041*
ACE of gender discrimination	1.1	0.7-2.0	0.645
Current exposure to spousal violence	2.5	1.5–4.3	0.001*
Older age of the mother	1.1	1.0-1.1	0.016*
Low socioeconomic status	0.7	0.3–1.9	0.486
Very low socioeconomic status	0.4	0.2–0.8	0.007*
High socioeconomic status	0.8	0.4–1.5	0.500
**Minor physical assault**			
ACE of physical abuse	1.5	0.7–3.1	0.292
ACE of emotional abuse	2.2	0.8–6.3	0.132
ACE of witnessing domestic violence	1.5	0.7-3.0	0.301
ACE of sexual abuse by an older person	1.0	0.3-3.0	0.990
ACE of gender discrimination	3.1	1.1–8.7	0.030*
ACE of divorced parents	1.2	0.3–4.2	0.773
ACE of household substance abuse	0.7	0.2-2.0	0.504
Current exposure to spousal violence	4.4	1.8–10.7	0.001*
Older age of the mother	1.2	1.1–1.2	0.001*
Low socioeconomic status	4.0	0.8–20.3	0.101
Very low socioeconomic status	1.4	0.6–3.3	0.431
High socioeconomic status	1.3	0.6–2.7	0.456
**Severe physical assault**			
ACE of physical abuse	1.5	0.8–2.8	0.191
ACE of emotional abuse	1.8	0.5–6.6	0.400
ACE of witnessing domestic violence	2.0	1.1–3.6	0.031*
ACE of gender discrimination	1.0	0.6–1.9	0.940
ACE of divorced parents	1.7	0.7–3.8	0.241
ACE of household substance abuse	1.8	0.8–3.7	0.137
Current exposure to spousal violence	2.6	1.5–4.6	0.001*
Older age of the mother	1.1	1.0-1.1	0.001*
Low socioeconomic status	1.4	0.5-4.0	0.574
Very low socioeconomic status	0.8	0.4–1.8	0.647
High socioeconomic status	0.8	0.4–1.6	0.476
**Very severe physical assault**			
ACE of physical abuse	1.8	0.9–3.6	0.114
ACE of emotional abuse	1.5	0.3–7.5	0.613
ACE of witnessing domestic violence	2.0	1.0-4.1	0.069
ACE of sexual abuse by an older person	1.4	0.6–3.1	0.460
ACE of gender discrimination	1.8	0.9–3.4	0.082
ACE of family member with chronic disease	1.7	0.6–4.6	0.296
Current exposure to spousal violence	2.6	1.4–4.8	0.002*
Older age of the mother	1.1	1.0-1.1	0.004*
Low socioeconomic status	9.5	2.4–36.2	0.001*
Very low socioeconomic status	3.3	1.1–10.0	0.031*
High socioeconomic status	2.6	0.9–7.5	0.078

OR^a^ odds ratio, CI^b^ confidence interval *Statistically significant at *p* < 0.05

Independent predictors of child ***neglect*** were an ACE of living with a family member with a mental health problem [OR = 3.4; 95% CI (1.1–10.7)], current exposure to spousal violence [OR = 2.5; 95% CI (1.5–4.3)], older age of the mother [OR = 1.1; 95% CI (1.0-1.1)] and very low socioeconomic status [OR = 0.4; 95% CI (0.2–0.8)].

Of the 12 variables associated with ***minor physical assault*** in the bivariate analysis, only ACE of gender discrimination [OR = 3.1; 95% CI (1.1–8.7)], mother’s current exposure to spousal violence [OR = 4.4; 95% CI (1.8–10.7)], and older age of the mother [OR = 1.2; 95% CI (1.1–1.2)] were found to be independent predictors.

***Severe physical assault*** was predicted by mothers’ ACE of witnessing domestic violence [OR = 2.0; 95% CI (1.1–3.6)], mothers’ current exposure to spousal violence [OR = 2.6; 95% CI (1.5–4.6)], and older age of the mothers [OR = 1.1; 95% CI (1.0-1.1)].

With respect to predictors of mothers’ perpetration of ***very severe physical assault***, mothers’ current exposure to spousal violence [OR = 2.6; 95% CI (1.4–4.8)], mothers’ older age [OR = 1.1; 95% CI (1.0-1.1)], low socioeconomic status [OR = 9.5; 95% CI (2.4–36.2)] and very low socioeconomic status [OR = 3.3; 95% CI (1.1–10.0)] were found to be significant independent predictors.

## Discussion

In the present study, mothers’ exposure to different ACEs during childhood was assessed and tested for associations with reported child abuse perpetration among their children. The percentages of mothers who had ever been exposed to ACEs and those with more than 3 ACEs were 66.3% and 18.6%, respectively. These rates were nearly in line with the rates reported in the first wave of the CDC-Kaiser ACE Study (64% and 25%) [27], and the rates reported by the Behavioural Risk Factor Surveillance System (BRFSS) study conducted in the USA (61.5% and 24.6%) [28]. 

On the other hand, studies from Arab countries revealed evidently higher rates of ever exposure as well as multiple exposures (> 2) to ACEs among Tunisian university female students (72.2% and 27.7%, respectively) [29] and adult Saudi women (80% and 55%) [13]. Differences between findings from Egypt and other Arab countries could be attributed to the cultural differences and community norms that accept violence as a positive family disciplinary practice, as well as methodological differences between studies, including sociodemographic characteristics of participants and assessment tools.

The current findings indicated that the most frequently reported ACEs were mainly related to family violence, psychological abuse, witnessing domestic violence, and physical and sexual abuse, followed by household substance abuse. These findings were in agreement with studies from the USA [28], Tunisia [15], and the Federation of Bosnia and Herzegovina [30], which pointed to emotional abuse/family violence as the most prevalent ACEs. Although there is partial agreement in the order of frequency of the different types of ACEs among different studies, the frequency of some types of ACEs varies according to the cultural background, prevailing social and parental practices and community norms of the study setting. This was evident in the findings of other studies that showed that the most common types were related to cultural practices not found in Egypt, such as living with anyone who was a problem drinker or alcoholic reported in the 2 waves of the Kaiser Study [22, 27], gender discrimination in Saudi Arabia [14], and parental death in Lebanon [31], which was attributed to war conflicts.

The present work provides further evidence that child abuse is a common parenting practice in successive generations in our community. Unfortunately, the offspring of the studied participants were not immune to childhood traumatic experiences. Psychological aggression was the most frequently reported type of child abuse perpetrated by sampled mothers, followed by corporal punishment, neglect, severe physical assault, and very severe physical assault. This order of frequency was comparable to the rates obtained from the Egypt UNICEF’s children survey in 2013 [11], the 2014 Egyptian demographic and health survey [7], and a review of a series of meta-analyses on child abuse [32]. 

Indeed, the higher rates of child abuse perpetrated by the studied women compared to that experienced by them during their childhood years do not necessarily reflect an actual increase in the prevalence of child abuse in our community over time. Recall bias in general and selective recall of only severe and traumatic experiences as well as differences in assessment tools and informants might explain these differences.

The findings of the current study revealed a statistically significant relationship between mothers’ childhood experiences of physical, psychological, or sexual abuse, as well as witnessing domestic violence and perpetration of different forms of child abuse toward their children. This finding provides evidence of the intergenerational transmission of violence among the studied mothers. Further evidence for this relationship was revealed through logistic regression analysis, where ACEs related to violence were found to be significant predictors of physical and psychological child abuse perpetrated by mothers.

Taken together, these findings support previous research evidence derived from studies from the USA [33] and KSA [34] as well as a meta-analysis by Madigan et al. on the intergenerational transmission of maltreatment, i.e., a parent’s history of maltreatment increases the risk of maltreatment among his/her children [33, 35]. 

Furthermore, the findings of the current study unveiled the dose‒response relationship between mothers’ ACEs and different types of child abuse in their offspring. This was clear from the statistically significant positive correlation that was found between maternal exposure to ACEs and the severity of perpetration of child corporal punishment as well as between maternal psychological abuse and neglect.

Similar findings were reported by Schofield et al. in 2018 [36] and Bartlett et al. in 2017 [37]. Moreover, type-specific transmission of child abuse was evident in mother’s ACE of psychological abuse that was a significant predictor of perpetration of the same type of abuse against her offspring, however this was not true for other types of abuse. In partial agreement, Sanchez and Berzenski et al. supported the evidence that the experience of a type of abuse was more likely to relate to that same type of abuse in the second generation than to other types; however, this was more pronounced in child physical abuse, which has commonly received wide interest from researchers than in other subtypes of child abuse [38, 39]. 

Importantly, physical child abuse in the present study was found to be predicted by other ACEs related to family violence (gender discrimination and witnessing domestic violence) rather than by a frank ACE of physical abuse. Gender discrimination and spousal violence are common practices in our community that put the exposed child at risk of perpetrating child abuse in adulthood.

The high rates of ACEs related to family violence among the studied women, as well as high rates of child abuse of women’s offspring, were compounded by the fact that approximately 1 in 3 women in the community are also victims of violence [40]. Furthermore, mothers’ victimization by husbands emerged as an independent predictor of all forms of child physical abuse as well as child neglect. Taken together, these findings suggest that family violence is a common experience among women in our community.

Evidence that children of young mothers are at increased risk of child maltreatment has been documented in several studies [7, 41–43]. This can be explained by the complex social and mental challenges young mothers face, including poverty, poor social support, an unmarried mother and maternal mental health problems. In contrast, the findings of the current study indicated that all child maltreatment types and neglect were predicted by older age of the mothers (≥ 30 years old). These findings can be attributed to the fact that almost all participants in the current study, irrespective of their age, had some protective factors, such as being married and being aged older at the time of the study or at the time of childbirth, were older than 20 years of age. Nevertheless, older mothers were more likely to have a longer duration of marriage, a greater number of children (up to 6 children), which constitute a large financial burden on low-income families and a source of stress that drains on women’s psychological reserves to tolerate child challenging behaviours in conflicting situations.

### Study limitations

Although the current study provides valuable information concerning the relationship between ACEs and perpetration of child abuse among mothers, several limitations exist. First, the study population was predominantly urban and was not representative of the majority of the Egyptian mothers. The participants were also selected from the FHCs with the highest attendance rate, which might predispose the sample to selection bias, limiting the generalizability of the study findings. Second, unlike longitudinal designs, cross-sectional designs limit follow-up assessments of abuse perpetration and causal inference. Third, the assessment of ACEs relied upon mothers’ reports of their childhood exposure, which might be associated with recall bias. Additionally, child abuse was evaluated based on mothers’ reports, which are subject to response bias, and mothers might not frankly report such practices, leading to underreporting of abuse rates. Methodological limitations related to the current study sample, such as participants’ sociodemographic characteristics and assessment tools, should be taken into consideration when comparing the current findings to those of other studies.

## Conclusions

The study findings indicated that ACEs, particularly those related to family violence, are prevalent among adult married women in our community. Collectively, the current study suggests that different forms of family violence, particularly child abuse and neglect and spousal violence, are strongly connected to each other in many important ways throughout different stages of women’s lives and across different generations. Indeed, time has come to address this complex problem through concerted national strategies across all levels and sectors, including health care, education, social affairs, mass media, and nongovernmental organizations. Multiple public health strategies and actions addressing violence and its determinants are recommended to target the problem at the national level. Educational strategies and awareness-raising actions through media campaigns are urgently needed to address the negative consequences of ACEs, the impact of violence on children and the promotion of nonviolent parenting skills and child discipline. The implementation of psychoeducational programmes for children and adolescents on how to manage and combat violence is ultimately important. In addition, identifying mothers and children exposed to abuse or violence and ensuring the provision of trauma-sensitive support services should be prioritized. Such services should include safe reporting mechanisms and psychological counselling. Further studies, including longitudinal studies and studies considering the child perspective, are recommended to expand our understanding of the interconnections among different forms of family violence.

### Electronic supplementary material

Below is the link to the electronic supplementary material.

**Supplementary Material 1: Appendix I.** Mothers’ Adverse Childhood Experiences

## Data Availability

The datasets used and analysed during the current study are available from the corresponding author upon reasonable request.

## Abbreviations

ACE(s): Adverse childhood experience(s)
CDC: Centres for Disease Control
CI: Confidence interval
CTSPC: Conflict Tactics Scales Parent‒Child
EDHS: Egyptian Demographic and Health Survey
FHC(s): Family Health Centre(s)
HITS tool: Hurt, Insult, Threaten, Scream tool
UNICEF: United Nations International Children’s Emergency Fund
USA: United States of America
