# Supplementary material for: The role of adverse childhood experiences in predicting child abuse perpetration among married mothers in Alexandria, Egypt: a cross-sectional study
Source: BMC Womens Health. 2024 Jan 23;24:59. doi: 10.1186/s12905-024-02903-9 (PMC10804716; doi:10.1186/s12905-024-02903-9)
Supplement: Supplementary file 1 — Supplementary Material 1: Appendix I. Mothers’ Adverse Childhood Experiences [file 12905_2024_2903_MOESM1_ESM.docx]

**Appendix I**

**Mothers’ Adverse Childhood Experiences**

| **During the childhood period some people are exposed to memorable situations and events that one couldn’t forget, to what extent have you faced any of the following:** | **Never** | **Seldom** | **Sometimes** | **Frequently** |
| --- | --- | --- | --- | --- |
| 1. Have you ever been physically hurt by a parent or other adult family member (e.g. hit, beat, slap, kick, burn) |  |  |  |  |
| 1. Have you ever been psychologically hurt by a parent or an adult family member (e.g., swear at you, insult you, put you down, threaten, shout). |  |  |  |  |
| 1. Did your parents ever behaved violently to one another up to an extend it emotionally hurt you (e.g., your father beating your mother). |  |  |  |  |
| 1. When you were a child, have you ever been forced by an adult or a person older than you for a sexual act (e.g., touched your body in a sexual way?) |  |  |  |  |
| 1. Have you ever felt that your parents treat your brother(s) better than you/preferred them that made you feel discriminated against. |  |  |  |  |
| **During your childhood:** | **Yes** | | **No** | |
| 1. Were your parents ever separated or divorced? |  | |  | |
| 1. Had any of your parents passed away? |  | |  | |
| 1. Was any of your household members mentally ill e.g., depressed, obsessive compulsive disorder, schizophrenia ...etc. |  | |  | |
| 1. Was any of your household members chronically ill with a severe disabling or killing disease (e.g. cancer) |  | |  | |
| 1. Have you ever lived with a family member who used illegal drugs (e.g. cannabis or addicting substances) |  | |  | |
| 1. Did a household member attempt suicide? |  | |  | |
| 1. Did a household member go to prison? |  | |  | |

1. No.1 Refers to the ACE of physical abuse
2. No. 2 refers to the ACE of emotional abuse
3. No. 3 refers to the ACE of witnessing domestic violence
4. No.4 refers to the ACE of sexual abuse by an older person
5. No.5 refers to the ACE of gender discrimination
6. No. 6 refers to the ACE of divorced parents
7. No. 7 refers to the ACE of a dead parent
8. No. 8 refers to the ACE of a family member with mental illness
9. No. 9 refers to the ACE of a family member with a chronic disease
10. No.10 refers to the ACE of household substance abuse
11. No. 11 refers to the ACE of a family member attempted suicide
12. No. 12 refers to the ACE of a family member imprisoned
